# Verification of Nux-Moschata

**Published:** 1897-08

**Authors:** J. C. White

**Affiliations:** Port Chester, N. Y.


### VERIFICATION OF NUX-MOSCHATA.

J. C. White, M. D., Port Chester, N. Y.

D. G., age twenty-six years, was admitted to the Women's Hospital of this village August 15th, 1896. Had been injured by the careless handling of a loaded gun, the charge entering the skull at the right parietal eminence or ridge, on a line with the auditory canal. Condition, partial collapse, though conscious; had motor paralysis of left upper limb and partial motor paralysis of left lower limb. Opening was enlarged to admit the finger, when the charge of small shot together with wadding was found near the surface of cerebrum, the fissures of which were destroyed to the depth and the diameter of one-quarter inch; part was cleansed of all foreign material and broken tissue and dressed antiseptically. Patient convalesced in about six weeks, and was able to leave the hospital with entire motor paralysis of the arm and slight impairment of the leg.

During his convalescence he suffered with severe headache, which was always better by hard pressure; wanted the nurse to "bear her whole weight on it." With this headache he had rapid and anxious respiration; seemed as if he could not get air enough in his lungs, and that his "wind would be shut off." These symptoms were very nicely relieved by Nux-mos. 3x; when the medicine was stopped for a day the pain in the head and rapid breathing would recur. Three several times I stopped the medicine with same result. I finally gave him a higher potency to use as required.

